# Angiotensin Type 2 Receptors: Painful, or Not?

**DOI:** 10.3389/fphar.2020.571994

**Published:** 2020-12-23

**Authors:** Lakshmi Pulakat, Colin Sumners

**Affiliations:** ^1^ Molecular Cardiology Research Institute, Tufts Medical Center, Boston, MA, United States; ^2^ Department of Medicine, Tufts University School of Medicine, Boston, MA, United States; ^3^ Department of Physiology and Functional Genomics, University of Florida, Gainesville, FL, United States

**Keywords:** neuropathic pain, angiotensin type 2 receptor, TRPA1, acute nerve injury, angiotensin type 2 receptors agonism, reactive oxygen species

## Abstract

Pain in response to various types of acute injury can be a protective stimulus to prevent the organism from using the injured part and allow tissue repair and healing. On the other hand, neuropathic pain, defined as ‘pain caused by a lesion or disease of the somatosensory nervous system’, is a debilitating pathology. The TRPA1 neurons in the Dorsal Root Ganglion (DRG) respond to reactive oxygen species (ROS) and induce pain. In acute nerve injury and inflammation, macrophages infiltrating the site of injury undergo an oxidative burst, and generate ROS that promote tissue repair and induce pain via TRPA1. The latter discourages using the injured limb, with a lack of movement helping wound healing. In chronic inflammation caused by diabetes, cancer etc., ROS levels increase systemically and modulate TRPA1 neuronal functions and cause debilitating neuropathic pain. It is important to distinguish between drug targets that elicit protective vs. debilitating pain when developing effective drugs for neuropathic pain. In this context, the connection of the Angiotensin type 2 receptor (AT_2_R) to neuropathic pain presents an interesting dilemma. Several lines of evidence show that AT_2_R activation promotes anti-inflammatory and anti-nociceptive signaling, tissue repair, and suppresses ROS in chronic inflammatory models. Conversely, some studies suggest that AT_2_R antagonists are anti-nociceptive and therefore AT_2_R is a drug target for neuropathic pain. However, AT_2_R expression in nociceptive neurons is lacking, indicating that neuronal AT_2_R is not involved in neuropathic pain. It is also important to consider that Novartis terminated their phase II clinical trial (EMPHENE) to validate that AT_2_R antagonist EMA401 mitigates post-herpetic neuralgia. This trial, conducted in Australia, United Kingdom, and a number of European and Asian countries in 2019, was discontinued due to pre-clinical drug toxicity data. Moreover, early data from the trial did not show statistically significant positive outcomes. These facts suggest that may AT_2_R not be the proper drug target for neuropathic pain in humans and its inhibition can be harmful.

## Introduction

**FIGURE 1:**
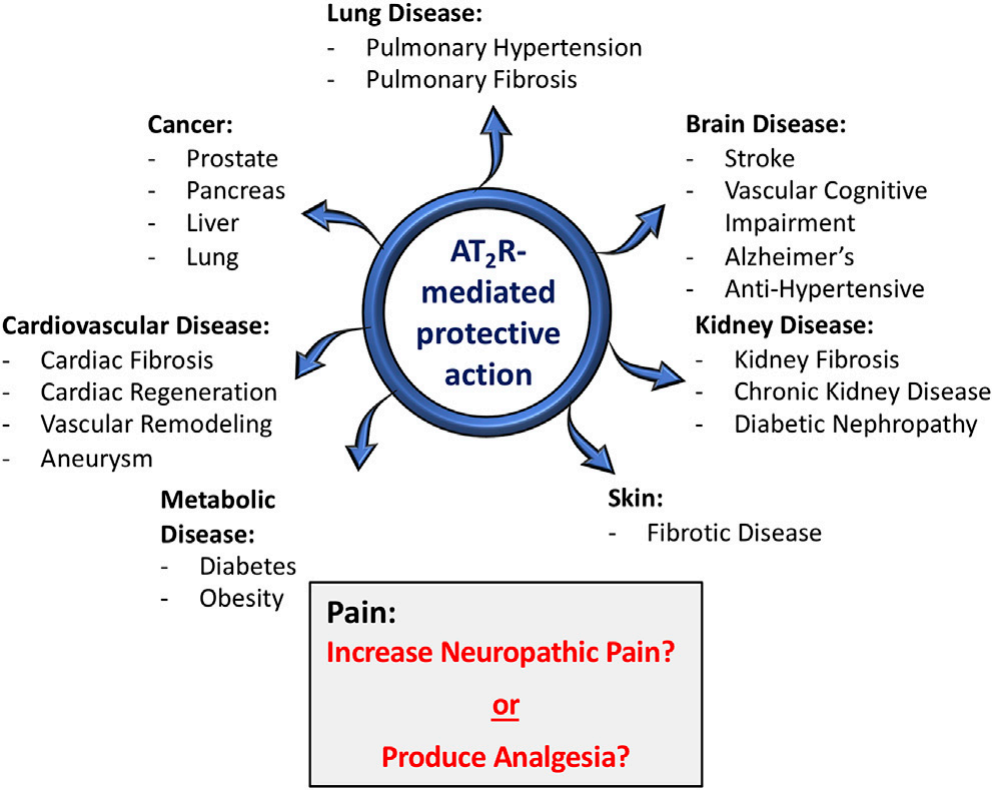
Role of AT_2_R in various disease conditions. In the vast majority of situations (blue arrows), AT_2_R agonist-induced activation or tissue over-expression of AT_2_R has been shown to exert protective actions in a host of disease conditions, particularly those with a strong inflammatory component. With regard to pain (gray shaded box), there are conflicting opinions, with some studies concluding that activation of AT_2_R exerts anti-nociceptive actions, while others conclude that AT_2_R antagonists produce relief from neuropathic pain.

Angiotensin type 2 receptors (AT_2_R), which were once considered to be a non-functional binding site for angiotensin II (Ang II), are now firmly established as one component of the “alternative” or “protective” renin-angiotensin system (RAS) (Unger et al., 2015). Along with angiotensin-(1-7) [Ang-(1-7)] and its receptor Mas, Ang II/AT_2_R constitute an arm of the RAS that for the most part exerts multiple beneficial actions at both the systemic and central levels, in various disease processes (Santos et al., 2019). The protective effects that result from the activation of AT_2_R and/or Mas can be independent of or are in opposition to the deleterious pathophysiological effects of Ang II via its well-known type 1 receptor (AT_1_R) (Santos et al., 2018; Santos et al., 2019). There is a great diversity of the beneficial effects of AT_2_R activation. For example, there are many studies which have demonstrated that AT_2_R agonists exert protective actions in fibrotic diseases (Wang et al., 2017; Sumners et al., 2019), in cardiovascular and renal diseases (Chow and Allen, 2016; Kaschina et al., 2017; Sharma et al., 2020) and there are multiple effects and beneficial disease implications for AT_2_R activation in the nervous system (Figure 1) (Guimond and Gallo-Payet, 2012; Sumners et al., 2013). However, despite the fact that a large majority of studies have projected AT_2_R activation as being “good” or beneficial, studies published within the past 5–6 years conclude that *blockade* of AT_2_R provides relief in neuropathic pain and inflammatory pain (Chakrabarty et al., 2013; Smith et al., 2013; Muralidharan et al., 2014; Shepherd et al., 2018). In contrast to this, other studies have implied that activation of AT_2_R can produce beneficial effects in neuropathic pain (Bessaguet et al., 2018). Nonetheless, the implication that AT_2_R activation is harmful under certain circumstances, i.e., it causes pain, has severe consequences for efforts that intend to take advantage of the beneficial action of AT_2_R agonists and translate findings in animal models to novel therapeutics for human disease. In this brief review the primary goal is to discuss the studies which have linked AT_2_R to pain, particularly with regard to the different types of pain, and to come up with an idea of whether this angiotensin receptor subtype is painful, or not. We begin with a brief review of what AT_2_R are, and their protective actions in disease states.

### Angiotensin Type 2 Receptors: Signaling and Function

Similar to the AT_1_R, the AT_2_R is a seven transmembrane domain receptor, and these receptors are 34% identical in terms of amino acid sequence and both contain most of the conserved motifs of a class A G-protein coupled receptor (GPCR) (Kambayashi et al., 1993; Mukoyama et al., 1993). But that is where the similarity between these receptors ends, and they differ greatly in terms of signaling, cellular actions and ultimately whole-body functions. The AT_1_R is well-known to signal through *G*_*q*_-mediated activation of phospholipase C (PLC) and increases in intracellular Ca^2+^ (Karnik et al., 2015). The AT_2_R certainly does not signal through the traditional GPCR signaling mechanisms such as activation of PLC/Ca^2+^ or modulation of cyclic AMP (Karnik et al., 2015). This might be explained by the findings of a crystallography study which indicate that helix VIII of the AT_2_R stabilizes the conformation of the receptor in its active state, at the same time covering the binding sites for G-proteins and β-arrestin, and thus preventing conventional GPCR signaling (Zhang et al., 2017). Nonetheless, functional studies have revealed that AT_2_R couple to several intracellular signaling mechanisms; these include both G-protein–and non-G-protein mediated pathways, and are often unique to the tissues in which the AT_2_R are located (Nouet and Nahmias, 2000; Sumners et al., 2019). For example, biochemical and functional studies have demonstrated that AT_2_R signal via an inhibitory G-protein and co-precipitate with G_i_ proteins (Kang et al., 1994; Hayashida et al., 1996; Zhang and Pratt, 1996; Hansen et al., 2000), and in neurons cultured from rodent brain AT_2_R-modulation of K^+^ currents and activation of serine-threonine phosphatase 2A (PP2A) occur via a pertussis toxin sensitive G-protein (Kang et al., 1994; Huang et al., 1995; Kang et al., 1995). On the other hand, AT_2_R can also activate tyrosine phosphatases, including Map Kinase Phosphatase-1 (MKP-1) and Src homology region two domain-containing-phosphatase-1 (SHP-1), and also phospholipase A2 (PLA_2_)/arachidonic acid (AA) pathways, albeit via non-G-protein-dependent mechanisms (Bedecs et al., 1997; Fischer et al., 1998). The activation of MKP-1, SHP-1 and also PP2A by AT_2_R certainly seem to interfere with kinase driven pathways, including Erk MAP kinase, providing a basis for anti-fibrotic actions of these receptors in a variety of tissues (Peluso et al., 2018; Sumners et al., 2019). Probably the most well-documented and robust signaling action associated with AT_2_R is the activation of endothelial nitric oxide synthase (eNOS), with subsequent generation of nitric oxide (NO) and cyclic guanosine monophosphate (cGMP) (Carey et al., 2000; Widdop et al., 2003). The induction of eNOS, mediated through activation of serine/threonine and tyrosine phosphatases, is of importance for certain anti-fibrotic and also vasodilatory actions of AT_2_R (Sumners et al., 2019). However, cell-specific differences in AT_2_R signaling is further highlighted by the observation that AT_2_R suppresses cGMP levels in oocytes, and this effect is regulated by the third intracellular loop of the AT_2_R and possibly involves an oocyte-specific SHP1-like protein (Pulakat et al., 2005). The third intracellular loop of AT_2_R is also a critical domain that interacts directly with AT_1_R to inhibit its signaling (AbdAlla et al., 2001; Kumar et al., 2002).

Unlike the dramatic physiological actions mediated by Ang II via AT_1_R, namely the maintenance of body fluid balance and blood pressure regulation, any effects of AT_2_R in *normal* animals are not as obvious and have been difficult to tease out. They are also quite diverse and don’t necessarily fit into a centralized theme, as do AT_1_R-mediated physiological effects. For example, activation of AT_2_R has been shown to stimulate natriuresis (Kemp et al., 2014), to inhibit vasopressin secretion (de Kloet et al., 2016), to lower blood pressure via a central mechanism (Steckelings et al., 2017), and to supress metabolism (Littlejohn et al., 2016). While these actions certainly occur, they are often moderate in nature, and they pale in comparison to the quite dramatic acts of AT_2_R in disease states, which will be discussed in the next section.

### Beneficial Actions of AT_2_R in Disease States

The functional effects of AT_2_R are far more profound in a variety of disease conditions. First of all, it is apparent that in comparison to AT_2_R levels in normal adult animals, the tissue expression of AT_2_R is greatly increased in many disease states, particularly those that involve inflammatory processes and tissue remodeling. Examples of these pathological conditions include: myocardial infarction, vascular injury, ischemic stroke, kidney failure, pulmonary fibrosis, skin wounds and sciatic or optic nerve transections (Booz and Baker, 1996; Steckelings et al., 2005; Jones et al., 2008; Lemarié and Schiffrin 2010). The increases in AT_2_R levels certainly appear to translate into functional effects, given the host of disease states in which there is abundant evidence that activation or increased expression of AT_2_R has been associated with beneficial effects. Probably the most profound effects of AT_2_R activation during disease conditions are the powerful anti-fibrotic actions in various lung-, cardiac-, vascular-, kidney- and skin diseases (Wang et al., 2017; Sumners et al., 2019). A major contributor to the potent anti-fibrotic effects of AT_2_R activation is significant anti-inflammatory actions (Rompe et al., 2010; Patel et al., 2020). In addition to anti-fibrotic actions, there exists much documentation of potent beneficial actions of AT_2_R activation in a variety of other disease processes, including diabetes (Paulis et al., 2016), obesity (Yvan-Charvet et al., 2005; Ali and Hussain, 2012), stroke (Bennion et al., 2018), vascular cognitive impairment (Mogi et al., 2012), aortic aneurysm (Verbrugghe et al., 2018; Sharma et al., 2020) and various cancers (Deshayes and Nahmias, 2005; Vinson et al., 2012). Female-specific increased expression of AT_2_R is implicated in protection from Ang II-induced increase in blood pressure and a reduction in AT_2_R expression in the heart tissues of female diabetic rats is associated with increased focal scarring in female rat heart (Sampson et al., 2008; Hilliard et al., 2012; Lum-Naihe et al., 2017). Since the beneficial actions of AT_2_R have been reviewed extensively elsewhere, for the present article we limit ourselves to only illustrating them in the diagram in Figure 1. It is worth pointing out, however, that in one case an AT_2_R agonist (Compound 21 [C21;VP01] Vicore Pharma, Gothenburg, Sweden) is undergoing clinical trials for idiopathic pulmonary fibrosis (https://vicorepharma.com/our-programs/pipeline/). Much more recently the same company has been approved for a clinical trial of C21/VP01 in patients infected with SARS-CoV-2, called ATTRACT (Angiotensin II Type Two Receptor Agonist Covid-19 Trial) (https://vicorepharma.com/our-programs/pipeline/). They believe that the potent anti-inflammatory effects of the AT_2_R agonist will be of benefit to patients with SARS-CoV-2 induced COVID-19. Thus, it is clear that the vast majority of cases, *activation* of AT_2_R exerts beneficial actions in disease states–hence the idea that these receptors are a component of the protective RAS. It is also clear that *activatio*n of these receptors presents a viable target for clinical development in a variety of diseases. However, as also seen in Figure 1, with regard to pain the story is different, with literature over the past 5–6 years concluding that AT_2_R *antagonists* are analgesic, particularly in neuropathic pain and chronic inflammatory pain (Smith et al., 2016). On the other hand, there are also studies which indicate that AT_2_R agonists exert beneficial effects in pain (Bessaguet et al., 2018).

If indeed AT_2_R *antagonists* are analgesic, then the implication is that activation of AT_2_R exerts pain, and that would have serious consequences for the further development of AT_2_R agonists for human diseases. The next section provides a detailed discussion of the role of AT_2_R in pain, with a view to understanding whether they are pro- or anti-pain, or both.

### Pain: Protective or Debilitating Stimulus in Acute Tissue Injury?

Last year, the International Association of the Study of Pain (IASP) proposed a new definition for pain in order to capture the current understanding of the ‘pain’. Accordingly, new definition of pain became “an aversive sensory and emotional experience typically caused by, or resembling that caused by, actual or potential tissue injury “(IASP Definition of Pain Task Force, 2019). Pain is induced by the activation of a subset of sensory neurons called nociceptors (Finnerup et al., 2016; Colloca et al., 2017; St John Smith 2018; Treede, 2018). The primary role of ‘pain’ during evolution is to detect dangerous stimuli that cause tissue damage and protect tissue from further damage (St John Smith 2018). Nociceptive pain can be categorized into radicular pain (where nerve roots are irritated due to conditions causing excess pressure or inflammation), somatic pain (where pain receptors in peripheral tissues such as muscle, skin, bone etc., are activated) and visceral pain (where internal organs such as the heart tissue is inflamed or damaged). Conversely, lesions or disease in the somatosensory system can cause chronic neuropathic pain that has no protective role and no effective treatment. Neuropathic pain can arise from damage to peripheral fibers (Aβ, Aδ and C fibers) and central neurons, and affects 7–10% of the general population. However, the understanding that the binary classification of pain purely as nociceptive or neuropathic leaves a good proportion of patients as unclassified. These patients have substantial overlap of nociceptive and neuropathic symptoms and this has resulted in coining of the term mixed pain (Freynhagen et al., 2019).

Chronic neuropathic pain is a debilitating condition and is more frequent in patients >50 years of age (8.9% compared to 5.6% in those <49 years of age), and in women compared to men (8% vs. 5.7% in men). Postherpetic neuralgia, trigeminal neuralgia, painful radiculopathy, diabetic neuropathy, HIV infection, leprosy, amputation, peripheral nerve injury pain and central post-stroke pain are all examples of neuropathic pain. Neuropathic pain (neuropathic pain) affects the lower back and lower limbs, neck and upper limbs, and is mechanistically different from chronic inflammatory pain such as that occur in rheumatoid arthritis where the primary cause is local chronic inflammation and resulting oxidative and nitrosative stress that irritate sensory neurons. However, neuropathic pain is associated with many chronic inflammatory conditions such as cancer, diabetes, multiple sclerosis, spinal cord injury, and in response to certain drug treatments such as chemotherapy (Finnerup et al., 2016; Colloca et al., 2017; St John Smith 2018; Treede, 2018; Freynhagen et al., 2019).

In 2008, a task force initiated by the IASP Special Interest Group on Neuropathic Pain (NeuPSIG) recognized the need to distinguish neuropathic pain from nociceptive pain arising indirectly from neurological disorders and pain conditions with secondary neuroplastic changes occurring in the nociceptive system. Their efforts resulted in the current definition of neuropathic pain as “pain caused by a lesion or disease of the somatosensory nervous system” (Treede et al., 2008; Finnerup et al., 2016). This definition allows considering the nociceptive pain conditions that over time cause secondary lesions in the somatosensory nervous system also as neuropathic pain. The NeuPSIG also developed a grading system to determine the certainty of neuropathic pain in clinical and research practices as ‘definite’, ‘possible ‘or ‘probable’ where the grade ‘possible’ does not affirm an neuropathic pain diagnosis, but just serves as a working hypothesis that pain may be categorized as neuropathic pain (Treede et al., 2008). The terms ‘probable’ and ‘definite’ require further neurologic examination.

Currently, neuropathic pain is an important socioeconomic health issue worldwide that affects millions of people and that does not have any effective treatments because of the wide variety of causes and signaling mechanisms that induce neuropathic pain. Thus, it is not surprising that the research community is constantly looking for new drug targets to curb neuropathic pain.

### TRPA1 in Neuropathic Pain

Calcium channels such as Transient Receptor Potential (TRP) channels (Patapoutian et al., 2009; Carrasco et al., 2018) are modulated by inflammation and overload of calcium ions, as well as oxidative and nitrosative stress resulting from inflammatory responses and immune system activation. TRP channels convert thermal and chemical stimuli into electrical activity on the peripheral terminals of sensory neurons. Recent studies have shown that members of the TRP subfamilies A (TRPA1), M (TRPM2 and 7), and V (TRPV1 and 4), in sensory neurons are involved in mediating nociception. The dorsal root ganglion (DRG) is an important neural structure in sensory transduction including pain transmission, and neuromodulation of persistent neuropathic pain (Berta et al., 2017; Deer et al., 2017; Esposito et al., 2019). Consistent with this role, DRG neurons exhibit high expression levels of TRPA1, TRPM2, TRPV1, and TRPV4 channels (Kobayashi et al., 2005; Deer et al., 2017; Carrasco et al., 2018; Esposito et al., 2019). Therefore, targeting DRG neuronal tissues and primary sensory neurons via gene and cell therapies as well as and peripheral pharmacological treatments are being developed to treat pain (Berta et al., 2017). TRPV1 is a well-established transducer of noxious stimuli and Story et al., showed that while 97% of TRPA1 (transient receptor potential ankyrin 1) expressing nociceptive neurons co-expressTRPV1, only 30% of TRPV1 expressing nociceptive neurons co-express TRPA1 (Story et al., 2003). Thus, TRPA1 is one transducer of pain pathways. TRPA1 serves as a common mediator for several chemically diverse molecules that function as pain and itch inducers including hydrophilic reactive oxygen and nitrogen species (ROS and RNS) induced by inflammatory responses, formalin, and pruritogens that mediate histamine-independent allergy-evoked itch, psoriasis and eczema. TRPA1’s ability to sense irritants is conserved from sea sponges to humans, suggesting an ancient origin (Kang et al., 2010). Pain response is attenuated by TRPA1 antagonists and in TRPA1 knock-out mice. All of these aspects have made TRPA1 as a primary target for pain therapy (Wilson et al., 2011; Chen and Hackos, 2015; Koivisto et al., 2018; Giorgi et al., 2019).

An elegant study by Trevisan et al. (2016) demonstrated that TRPA1 is a mediator for the Trigeminal neuropathic pain in response to oxidative stress induced by oxidative/nitrosative burst of monocytes/macrophages. In this study, C57BL/6 and wild-type (TRPA1 (+/+)) mice that were subjected to constriction of the infraorbital nerve exhibited significant prolonged non-evoked nociceptive behavior and mechanical, cold and chemical hypersensitivity compared to sham-operated mice and these pain-like behaviors were abated by genetic deletion or chemical inhibition of TRPA1, anti-oxidants, and inhibition of increases in monocytes/macrophages. Based on their findings they proposed that in the infraorbital nerve constriction model of trigeminal neuropathic pain, oxidative stress by-products released from monocytes and macrophages accumulate at the site of nerve injury and activate TRPA1 channels to cause pain-like behavior (Trevisan et al., 2016). Moreover, De Logu et al. (2017) demonstrated that TRPA1 channels in Schwann cells are involved in neuroinflammation and activation of NADPH oxidase 1 (NOX1)-dependent hydrogen peroxide release. They also showed that inhibiting this effect attenuated macrophage infiltration. Additionally, macrophages recruited to the perineural space underwent NOX2-dependent oxidative burst and further activated the TRPA1-NOX1 pathway in Schwann cells, but not TRPA1 in nociceptors. The authors concluded that Schwann cell TRPA1 activates a spatially constrained gradient of oxidative stress to sustain continuous macrophage infiltration to the injured nerve and activate TRPA1 nociceptors, causing mechanical allodynia via a paracrine mode of action.

The role of nitroxidative signaling in pain has been studied in many different rodent models for inflammatory pain and neuropathic pain. Extensive research shows that nitroxidative species generated by mitochondria as well as by NADPH oxidase and nitric oxide synthase enhance neuroexcitability to sustain pain through direct neuronal interactions, and indirectly by impairing mitochondria and inducing neuroinflammation (Grace et al., 2016). However there are several unanswered questions regarding the ubiquitous nature, species specificity, and roles of anti-oxidants and anti-inflammatory signaling pathways in regulating nitroxidative signaling in pain (Grace et al., 2016). These questions are particularly relevant to the question of whether AT_2_R is an inducer of pain or not. This is because AT_2_R is an inhibitor of NADPH oxidase and generation of ROS in different cell types including neuronal cells (Lu et al., 2015; Toedebusch et al., 2018; Bhat et al., 2019).

Several studies showed that TRPA1 is a thermosensor. In invertebrates and ancestral vertebrates (fly, mosquito, frog, lizard and snakes) TRPA1 serves as a heat receptor that induces avoidance of heat and infrared detection (Laursen et al., 2015) whereas in mammals it mediates cold hypersensitivity (Obata et al., 2005; Sawada et al., 2007; del Camino et al., 2010; Chen et al., 2013). However, a study using four different mammalian species (mouse, rat, rhesus monkey and human) showed that TRPA1’s cold hypersensitivity is specific to rodents, but not primates, making the translational significance of cold hypersensitivity studies in rodents weak (Heber and Fischer, 2019). Moreover, although TRPA1 antagonists (A–967079), show positive results in rodent neuropathic pain studies, they have not been validated for therapeutic use due to limited efficacy in the chosen models, or issues during development of the drug (Heber and Fischer, 2019). Recent studies have indicated TRPA1 plays a central role in Ang II-induced cold hypersensitivity in mice.

### Angiotensin II and TRPA1

Shepherd et al. (2018), showed that in a spared nerve injury (SNI) mouse model Ang II levels were elevated in the ipsilateral sciatic nerves and Ang II injection into mouse hind paws induced mechanical hypersensitivity in a dose-dependent manner. While blockade of the AT_1_R with losartan did not modulate Ang II-induced mechanical hypersensitivity, co-administration of the AT_2_R antagonist PD123319 attenuated both SNI-induced and Ang II-induced mechanical hypersensitivity in a dose-dependent manner and to similar extents in both sexes. However, intrathecal (i.t.) administration of PD123319 did not attenuate mechanical hypersensitivity, indicating that AT_2_R is not directly involved in this type of acute mechanical injury-associated pain mediated via DRG neurons and in addition there was no detectable AT_2_R mRNA or protein expression in mouse or human sensory neurons (Shepherd et al., 2018). Additionally, hind paw injection of Ang II did not induce mechanical hypersensitivity in this study. Further examination showed that systemic administration of TRPA1 inhibitors attenuated Ang II-induced mechanical hypersensitivity. Similar results were obtained for Ang II- or SNI-induced cold hypersensitivity. These observations strongly suggested that Ang II-induced mechanical and cold hypersensitivity in mice is mediated via TRPA1. However, prolonged exposure of mouse and human DRG neurons to Ang II did not induce calcium overload or TRPA1 activation nor modulate action potential firing or other membrane potential properties of these neurons. These observations strongly suggest that the Ang II-induced mechanical and cold hypersensitivity that are indicators of neuropathic pain are not mediated by neuronally-located AT_2_R. Moreover, it is also noteworthy that TRPA1-mediated cold hypersensitivity is not observed in humans and therefore, this AT_2_R-effect in mice is not translatable (Heber and Fischer, 2019).

### Oxidative Burst by Macrophages at the Site of Acute Tissue Injury and Tissue Repair–Is it a Protective or a Debilitative Pain Stimulus

**FIGURE 2:**
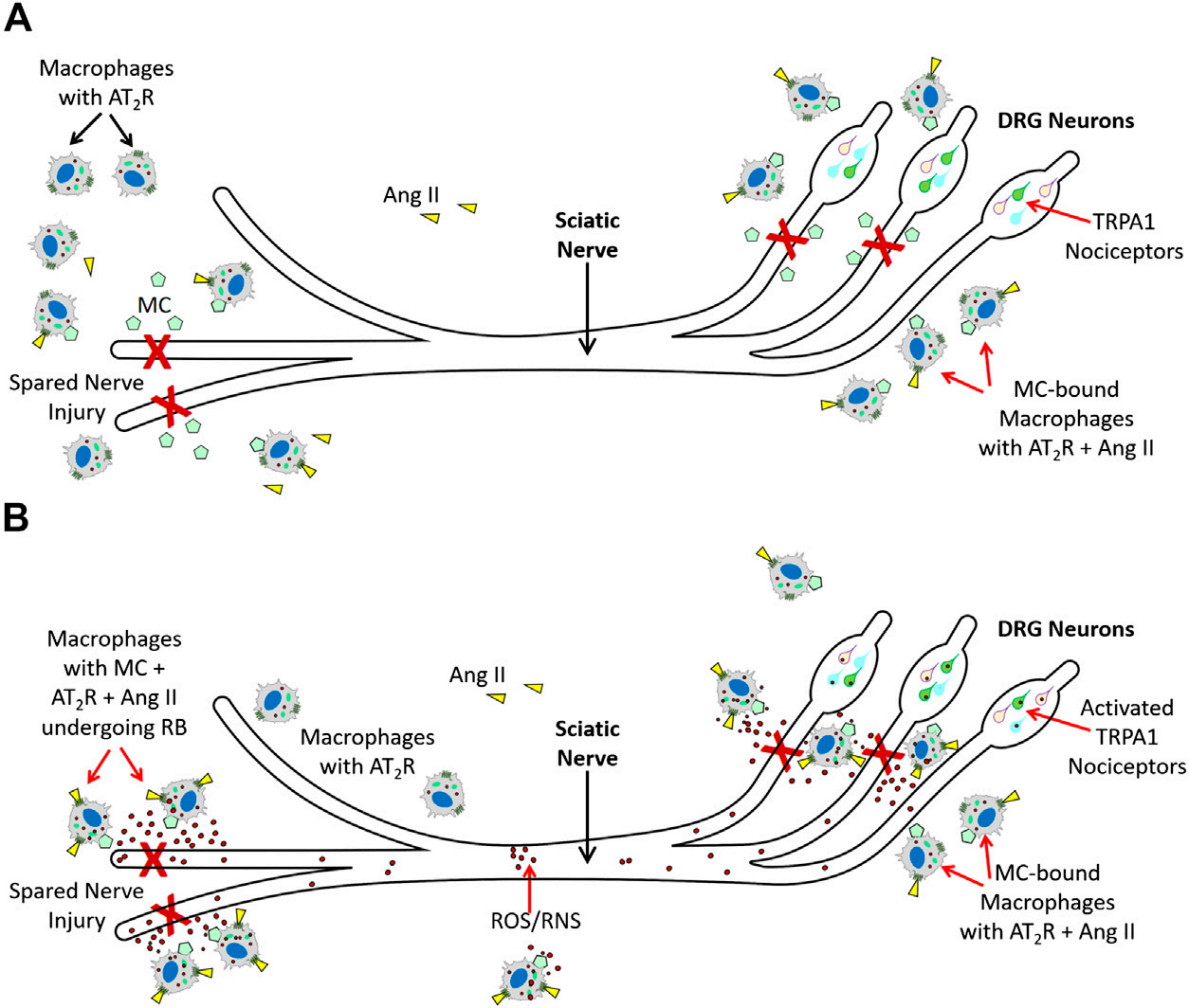
Molecular mechanisms AT_2_R-mediated nociception **(A)**. Spared Nerve Injury (experimental injury) in mice activates tissue RAS, and increases Ang II. Ang II (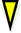) binds to AT_2_R (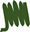) on macrophages. At the same time macrophages are attracted to the injury site by macrophage chemoattractants (MC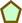). Those macrophages that interact with MC are directed to injured tissue. (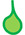): TRPA1 Nociceptor in DRG. **(B)**. Macrophages with AT_2_R (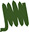) at the injured site bind Ang II (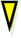) and undergo respiratory burst (RB), an innate immune response that is an integral part of removal of any pathogens, and activates macrophage erythropoietin signaling to promote acute inflammation resolution. According to Shepherd *et al.*, reactive oxygen/nitrogen species (ROS/RNS [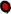]) generated during this process activates TRPA1 nociceptors (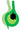) in DRG and cause pain. Since AT_2_R is a reparative/wound healing molecule, its involvement in activating respiratory burst of macrophages, an essential mechanism for inflammation resolution, and thereby indirectly activating TRPA1 nociceptors is part of a protective pain mechanism rather than debilitating chronic neuropathic pain.

Shepherd et al. (2018), further showed that Ang II-induced TRPA1 activation in sensory neurons is an indirect mechanism and requires peripheral macrophage-induced redox activation that increases ROS/RNS in response to Ang II. TRPA1 on the sensory neurons was responding to exposure to ROS/RNS produced by these macrophages, and AT_2_R present on the macrophages promoted redox activation in response to Ang II. Importantly, peripheral macrophages did not express any functional TRPA1. Thus, macrophage AT_2_R also did not directly activate TRPA1. While these observations have unveiled an elegant immune cell-neuronal cell communication via ROS/RNS using indicators such as cold and mechanical hypersensitivity in an SNI mouse model, it is important to note that this is not an example of chronic neuropathic pain in humans. In an acute injury mouse model such as SNI model, the role of invading macrophages is to mediate an oxidative/nitrosative burst to destroy any pathogens at the injured site, and help in tissue repair. The oxidative burst by macrophages at the site of injury is required for promoting tissue repair and is a necessary process for healing rather than a debilitating pathological process (Santabárbara-Ruiz et al., 2015; Yang et al., 2019). Therefore, we propose that TRPA1 activation on the sensory neurons is elicited by ROS/RNS coming from the invading macrophages (Figure 2) and subsequent induction of pain serves as a protective stimulus in an acute injury SNI mouse model to reduce limb movement and expedite the healing process, rather than a pathological stimulus that causes chronic debilitating neuropathic pain.

Several studies have shown AT_2_R activation actually inhibits oxidative stress and reduces ROS levels. For example, CGP42112, an AT_2_R agonist, is shown to suppress oxidative stress and NADPH oxidase (NOX) expression in a rotenone model for Parkinson's Disease in CATH.a cells (Lu et al., 2015). In microglia, AT_2_R activation is shown to inhibit NOX activation, ROS production, and pro-inflammatory microglia activation, and promote immunoregulatory microglia. These protective effects of AT_2_R involve the protein phosphatase 2A (PP2A)-mediated inhibition of protein kinase C (PKC), that prevents the NOX activation, ROS generation, and subsequent pro-inflammatory activation of microglia (Bhat et al., 2019). In human coronary vascular smooth muscle, another AT_2_R agonist, NP-6A4 has been shown to suppress Doxorubicin-induced increase in cellular ROS (Toedebusch et al., 2018). These observations raise the question that if AT_2_R activation in neuronal cells and microglia reduces ROS levels and protects these cells from ROS/RNS-induced injury, will AT_2_R activation in these cells attenuate ROS-induced TRPA1 activation?

### AT_2_R-Mediated Nociceptive- and Anti-Nociceptive Actions: A Summary of Further Studies

Aside from the studies of Shepherd et al., 2018, a number of other studies performed during the past 20–30 years have indicated nociceptive–or anti-nociceptive actions of AT_2_R. These are summarized in Table 1, and certain of them are discussed in more detail below.

**TABLE 1:** Studies that imply Nociceptive - or Anti-Nociceptive roles for AT_2_R in pain.

AT_2_R action	Animal model/Tissues affected	Nerve injury	Drug dose/Route or receptor manipulation	Outcome	Mechanism	References
**Nociceptive**	Sciatic nerve injury (rat, mouse)/hind-paw hypersensitivity	Unilateral chronic constriction injury (CCI) of the sciatic nerve	EMA200, EMA300, EMA400 (1–10 mg bolus) - IP	AT_2_R antagonists elicited analgesia (EMA400 > EMA300 > EMA200)		Smith et al. (2013)
		CCI of the sciatic nerve - hind paw hypersensitivity	EMA300 (10 mg/kg) - IP	AT_2_R antagonist elicited analgesia	Inhibition of CD3^+^ T cell infiltration and increase in nerve growth factor NGF	Khan et al. (2017)
		Rodent hind paw model of inflammatory pain: thermal and mechanical hypersensitivity	PD123319 (5 mg/kg/day) - IP infusion PD123319 (10 mg/kg bolus) - IP	Inhibits nociceptor hyperinnervation and hypersensitivity inhibits thermal hypersensitivity	Prevents nociceptor hyperinnervation associated with inflammatory pain	Chakrabarty et al. (2013)
		SNI-induced peripheral neuropathy: Mechanical or cold-induced hypersensitivity	PD123319 (10 mg/kg) - IP	AT_2_R antagonist attenuated mechanical or cold-hypersensitivity	Macrophage AT_2_R promote ROS/RNS production and subsequent activation of TRPA1 on sensory neurons	Shepherd et al. (2018)
		SNI-induced peripheral neuropathy: Mechanical or cold-hypersensitivity	PD123319 (10 mg/kg) - IP	AT_2_R antagonist elicited analgesia		Shepherd and Mohapatra (2019)
		****	Clinical trial: Post -herpetic neuralgia	Skin and nerve fibers	EMA401 (100 mg, twice daily) - PO	AT_2_R antagonist provides superior relief of pain associated with postherpetic neuralgia		Rice et al. (2014)
			Clinical trial: Post -herpetic neuralgia	Skin and nerve fibers	EMA401 (25 mg, or 100 mg, or 300 mg, twice daily)	Study was prematurely terminated. No positive outcomes and pre-clinical toxicity data	_	https://clinicaltrials.gov/ct2/show/NCT03094195
				Rat model of prostate cancer-induced bone pain	Hind-paw hypersensitivity	EMA200 (0.3-10 mg/kg bolus) - IV	AT_2_R antagonist elicited dose-dependent analgesia	Decreased Ang II level, increased NGF/trkA signaling, inhibition of p38- and Erk MAP kinase activation	Muralidharan et al. (2014)
			**Anti-Nociceptive**	Rat model for acetic acid-induced abdominal writhing –chemical injury	Abdominal muscles and nerves	Ang II (0.05–1.0 μg bolus) - ICV ± PD123319 (10 μg bolus) -ICV ± los (25 μg bolus) - ICV	Ang II- induced anti-nociception blocked by AT_2_R antagonist, not by AT_1_R antagonist		Georgieva and Georgieva (1999)
	Mouse model for tail flick and tail-pinch tests	Tail tissue and nerve fibers		AT_2_R-deletion	Pain threshold significantly lower in AT_2_R-deficient mice	Decreased levels of β-endorphin in brain	Sakagawa et al. (2000)
Mouse model for *Mycobacterium ulcerans* infection or mycolactone injection	Foot pads for infection and tail flick assay; tail tissue, nerve fibers and *in vitro* studies on PC12 neurons	Mycolactone acts as AT_2_R agonist		Pain threshold significantly lower in mice exposed to mycolactone	Mycolactone through AT2R leads to potassium-dependent hyper-polarization of neurons	Marion et al. (2014)
Vincristine (VCR)-induced neuropathy in mice; mechanical allodynia	Non-peptidergic intraepidermal nerve fibers; myelinated nerve fibers in the sciatic nerve	Compound 21 (0.3 mg/kg/day, for 16 days) - IP		AT_2_R agonist restored normal mechanical sensitivity in VCR-treated mice	Prevention of non-peptidergic C fiber loss; protection against VCR-induced loss and enlargement of myelinated nerve fibers in the sciatic nerve	Bessaguet et al. (2018)

Key: Ang II = angiotensin II; PD123319, EMA200, EMA300, EMA400, EMA401 are all AT_2_R antagonists; C21, AT_2_R agonist; IP, intraperitoneal; IV, intravenous; PO, oral; ICV, intracerebroventricular.

Two early studies indicated an anti-nociceptive role of AT_2_R. First, it was shown 2 decades ago that Ang II-mediated activation of brain AT_2_R is anti-nociceptive in a mouse model of pain induced by acetic acid-induced abdominal constriction (Georgieva and Georgiev, 1999). In this model, abdominal constrictions were counted at 5-min intervals for 30 min and intracerebroventricular (ICV) administration of Ang II at doses of 0.05, 0.1, and 1 microg exerted a dose-dependent anti-nociceptive effect. PD123319 (also delivered via ICV) suppressed these Ang II-induced anti-nociceptive effects while losartan had no effect. The authors concluded that the Ang II-induced anti-nociceptive effect required AT_2_R signaling in this model.

A second study examined whether AT_2_R influences pain threshold. Sakagawa et al. (2000) used AT_2_R deficient mice and demonstrated that pain threshold was significantly lower in these animals compared to wild type mice. They also found that AT_2_R deficiency did not modulate learning behavior and brain edema formation, but AT_2_R deficient mice had lower levels of β-endorphin in the arcuate nucleus of the medial basal hypothalamus (ARC) compared to wildtype mice. Moreover, they did not find any differences between AT_2_R deficient mice and wild type mice in passive avoidance task and cold injury indicating that lack of AT_2_R did not provide any advantage in handling these types of pain.

A more recent study suggests a nociceptive action of AT_2_R. In that study, it was demonstrated that another AT_2_R antagonist, EMA300, inhibits peripheral neuropathic pain, again not via effects on neuronal AT_2_R function (Khan et al., 2017). EMA 300 and 401 are small compounds that have high selectivity for the AT_2_R (>1000-fold binding selectivity for the AT_2_R over AT_1_R). In a randomized, double-blind, placebo-controlled clinical trial that involved 183 patients with post herpetic neuralgia, twice-daily oral administration of EMA401 that cannot enter the brain evoked significant analgesia (Rice et al., 2014). To understand the molecular mechanisms underlying anti-neuropathic pain effects of the EMA compounds, Khan et al., investigated the effects of a single intraperitoneal bolus dose of EMA300 (10 mg/kg), or vehicle on unilateral hindpaw hypersensitivity in rats following chronic constriction injury (CCI) of the sciatic nerve. Similar to the 2018 Shepard et al., study, these authors also found a significant increase in Ang II levels in the injured (ipsilateral) DRG neurons. They also found an increase in CD3^+^ T cell infiltration in the vehicle treated ipsilateral lumbar DRGS that contributed to the increase in Ang II levels, and this infiltration was suppressed by EMA300 treatment. Importantly, they did not find any changes in the expression levels of AT_2_R in vehicle treated ipsilateral DRG neurons. The authors concluded that the effect of EMA300 on pain reduction is mediated via inhibition of CD3^+^ T cell infiltration and an increase in nerve growth factor NGF. However, it is unclear whether these Ang II/AT_2_R expressing CD3^+^ T cells were actually cytotoxic in this study. This question is very relevant considering that there is evidence that shows infiltration of AT_2_R expressing CD8^+^ T cells to injured tissue does not have to be cytotoxic. For example, during ischemic heart injury there is an increase in infiltration of AT_2_R expressing CD8^+^ T cells. Activation of AT_2_R in these cells contributed to IL-10 production and intramyocardial transplantation of CD8^+^ AT_2_R + T cells actually reduced ischemic heart injury (Curato et al., 2010). Therefore, additional studies are needed to understand the exact role (pro-inflammatory or anti-inflammatory) of Ang II/AT_2_R expressing CD3^+^ T cell infiltration in this CCI injury site.

It is important to note that activation of p38 mitogen-activated protein kinase (MAPK) and phosphorylated-p44/p42 MAPK as well as Erk1/2 can occur in response to Ang II mediated via AT_1_R (Wei et al., 2008; Xiao et al., 2013; Nemoto et al., 2015). In the studies using PD123319 and EMA300, activation of these pathways in sensory neurons in response to SNI, CCI, or Ang II injection were observed and their inhibition by either PD123319 or EMA300 was considered as evidence that these pathways are activated by AT_2_R. Both PD123319 and EMA300 only have increased selectivity to AT_2_R, not an absolute lack of affinity to AT_1_R. Thus, in the absence of antagonists for AT_2_R that absolutely do not bind AT_1_R even at higher doses, it is difficult to say whether the observed effects of these drugs on immune cell infiltration and subsequent reduction in Ang II levels at the site of nerve injury that attenuates mechanical or cold hypersensitivity is actually mediated via AT_1_R or AT_2_R inhibition. Additionally, it has been demonstrated that p38 MAPK activation mediated through AT_1_R on spinal astrocytes and neurons is involved in Ang II- And III-induced nociceptive behavior in mice and this effect was inhibited by losartan and p38 MAPK inhibitor SB203580, but not by the AT_2_R antagonist PD123319, the MEK1/2 inhibitor U0126 or the JNK inhibitor SP600125 (30). Moreover, the clear evidence that AT_2_R agonism suppresses oxidative stress (ROS/RNS) in neurons and glia that can prevent TRPA1 activation, and that neither DRG-neuronal nor peripheral-macrophage AT_2_R activate TRPA1 (the mediator of mechanical and cold hypersensitivity) in these cells, questions whether AT_2_R is actually a pain inducer for neuropathic pain. This contention is supported by recent studies which demonstrate that the non-peptide AT_2_R agonist Compound 21 is protective against vincristine-induced neuropathic pain (Bessaguet et al., 2018). The same group had earlier demonstrated that the beneficial effects afforded by the AT_1_R blocker candesartan in resiniferatoxin-induced neuropathic pain were due to generation of Ang II and stimulation of the AT_2_R (Bessaguet et al., 2017).

It is important to note that Novartis Pharmaceuticals acquired the AT_2_R antagonist EMA401 through its US$200 million acquisition from Spinifex Pharmaceuticals in 2015 to develop it as a drug for indications including Diabetic neuropathies, Neuropathic pain, and Post-herpetic neuralgia. Its brand name is Olodanrigan and if it could have been successfully commercialized, the deal could have topped over $1billion. Thus, Novartis initiated new clinical trials to validate the protective effect that this AT_2_R antagonist had previously showed in the Rice et al. (2014) paper. However, according to the new reports in 2020, Novartis has discontinued the drug (https://adisinsight.springer.com/drugs/800022957; June 10, 2020 update: https://biotechdispatch.com.au/news/disappointment-for-australian-innovation). The data posted on the clinical trial site for ‘Dose Response Study of EMA401 in Patients With Post-herpetic Neuralgia (PHN) (EMPHENE)’ (https://clinicaltrials.gov/ct2/show/NCT03094195, last update posted May 14, 2020) states that patient recruitment was “Terminated (The study was terminated early due to pre-clinical toxicity data that became available after start of trial)”. The study was supposed to use three doses of EMA401 (25, 100 or 300 mg) administered twice daily via oral delivery. They completed the 25 and 100 mg studies along with placebo in a total of 129 participants. Thus far, data (with *p* values) has been provided for two outcome measures The outcome measure on ‘Change in Weekly Mean 24-h Average Pain Score Using the 11 Point Numerical Rating Scale (NRS) From Baseline to Week 12’ did not show significant statistical difference for either 25 mg or 100  mg doses compared to placebo (*p* values 0.689 and 0.350 respectively). The outcome measure on ‘Percentage of Patients Achieving at Least 30% Pain Reduction at Week 12 on NRS 11 Point Scale’ also did not show significant statistical difference for either 25 or 100 mg compared to placebo (*p* values 0.908 and 0.609 respectively). Countries participating in the study were Australia, Austria, Belgium, Canada, Czech Republic, Denmark, France, Germany, Hungary, Italy, Japan, South Korea, Norway, Poland, Portugal, Slovakia, Taiwan, United Kingdom, Spain. These new developments regarding the safety and efficacy of EMA401 and its potential as a drug for neuropathic pain are not in accordance with its expected protection from neuropathic pain in humans.

Conversely, these results may not be surprising considering the protective effects of AT_2_R in many tissues as described in previous sections, and the loss of such protection resulting from inhibition of AT_2_R signaling induced by the systemically administered AT_2_R antagonist. In this context, it is noteworthy that loss of AT_2_R expression due to the intronic G1675A or A1818T polymorphism in men is associated with impaired kidney function, pulse pressure, and increased arterial stiffness (Pettersson-Fernholm et al., 2006; Cwynar et al., 2016). Thus, existing human data indicate that in humans systemic suppression of AT_2_R is not a safe approach.

Given the conflicting data on the ability of EMA401 to protect humans from PHN, the question remains whether AT_2_R is actually the appropriate target for neuropathic pain in humans. Conversely, *Mycobacterium ulcerans* induces severe lesions (Buruli Ulcer) without pain in humans. Pre-clinical studies show that mycobacterial polyketide mycolactone is responsible for this phenomenon and this effect is achieved by mycolactone-induced activation of AT_2_R leading to potassium-dependent hyperpolarization of neurons that induces analgesic effects (Marion et al., 2014). Collectively, these observations argue against AT_2_R’s role in causing neuropathic pain.

## Conclusions

There are conflicting reports as to the role and activity of AT_2_R in pain, with some studies concluding that AT_2_R are pro-pain, while others conclude that AT_2_R are anti-pain. The picture on the role of AT_2_R in pain is likely muddied by the fact that pain itself is not straightforward–on the one hand it can be protective in terms of discouraging an individual not to use (for example) an injured limb or other body part, but on the other hand it can be debilitating neuropathic pain without protective value. In the context of AT_2_R and pain, many of the studies which concluded that AT_2_R antagonists are beneficial in neuropathic pain utilize acute nerve injury or inflammatory pain models that are different from and do not mimic chronic neuropathic pain. Moreover, the recent clinical trial EMPHENE by Novartis Pharmaceuticals to test the effect of Olodanrigan (AT2R antagonist EMA401) on neuropathic pain in PHN patients was prematurely terminated due to additional pre-clinical data indicating drug toxicity. Additionally, available clinical trial data did not show any statistically significant positive outcomes after 12-weeks of treatment. These observations challenge the concept that AT_2_R antagonism protects humans from neuropathic pain. They bring up two critical points: 1) We currently have no conclusive evidence that supports AT_2_R antagonism prevents neuropathic pain in humans; 2) AT_2_R antagonism with EMA401 is not a safe treatment. Another important point to consider is the AT_2_R’s ability to suppress ROS levels in cells. While AT_2_R activation in macrophages causes an oxidative/nitrosative burst, and subsequent ROS/RNS-induced TRPA1 activation causes pain in an acute nerve injury, it is unclear whether AT_2_R-mediated signaling in other AT_2_R expressing cells contributes to ROS suppression and how the AT_2_R-induced ROS suppression modulates pain. From our review of this area we propose that the AT_2_R does not seem to be the inducer of neuropathic pain in humans. Moreover, since AT_2_R activation suppresses ROS in neuronal cells expressing AT_2_R and mouse DRG neurons do not express AT_2_R, additional studies are warranted to validate that an AT_2_R-ROS-TRPA1 pathway for nociception is actually conserved in humans.

## Author Contributions

Both authors listed wrote the manuscript, edited the document and approved it for publication.

## Funding

This work was supported by NIH R01HL138988 (LP) and NIH R01 HL136595 (CS).

## Conflict of Interest

The authors declare that the research was conducted in the absence of any commercial or financial relationships that could be construed as a potential conflict of interest.

## References

[B1] AbdAlla S. Lother H. Abdel-tawab A. M. Quitterer U. (2001). The angiotensin II AT2 receptor is an AT1 receptor antagonist. J. Biol. Chem. 276 (43), 39721–39726. 10.1074/jbc.M105253200 11507095

[B2] Ali Q. Hussain T. (2012). AT_2_ receptor: Its role in obesity associated hypertension. Int. J. Clin. Pharmacol. Toxicol. 1 (1), 15–19. 10.19070/2167-910X-120003 27588222 PMC5004927

[B4] Bedecs K. Elbaz N. Sutren M. Masson M. Susini C. Strosberg A. D. (1997). Angiotensin II type 2 receptors mediate inhibition of mitogen-activated protein kinase cascade and functional activation of SHP-1 tyrosine phosphatase. Biochem. J. 325 (Pt 2), 449–454. 10.1042/bj3250449 9230127 PMC1218581

[B5] Bennion D. M. Steckelings U. M. Sumners C. (2018). Neuroprotection via AT(2) receptor agonists in ischemic stroke. Clin. Sci. 132 (10), 1055–1067. 10.1042/CS20171549 29802210

[B6] Berta T. Qadri Y. Tan P. H. Ji R. R. (2017). Targeting dorsal root ganglia and primary sensory neurons for the treatment of chronic pain. Expert Opin. Ther. Targets 21 (7), 695–703. 10.1080/14728222.2017.1328057 28480765 PMC5890331

[B7] Bessaguet F. Danigo A. Bouchenaki H. Duchesne M. Magy L. Richard L. (2018). Neuroprotective effect of angiotensin II type 2 receptor stimulation in vincristine-induced mechanical allodynia. Pain 159 (12), 2538–2546. 10.1097/j.pain.0000000000001361 30086116

[B8] Bessaguet F. Danigo A. Magy L. Sturtz F. Desmoulière A. Demiot C. (2017). Candesartan prevents resiniferatoxin-induced sensory small-fiber neuropathy in mice by promoting angiotensin II-mediated AT2 receptor stimulation. Neuropharmacology 126, 142–150. 10.1016/j.neuropharm.2017.08.039 28882562

[B9] Bhat S. A. Sood A. Shukla R. Hanif K. (2019). AT_2_R activation prevents microglia pro-inflammatory activation in a NOX-dependent manner: inhibition of PKC activation and p47phox phosphorylation by PP2A. Mol. Neurobiol. 56 (4), 3005–3023. 10.1007/s12035-018-1272-9 30076526

[B10] Booz G. W. Baker K. M. (1996). The role of the renin-angiotensin system in the pathophysiology of cardiac remodeling. Blood Pres Suppl. 2, 10–18. 8913534

[B11] Carey R. M. Jin X. Wang Z. Siragy H. M. (2000). Nitric oxide: a physiological mediator of the type 2 (AT2) angiotensin receptor. Acta Physiol. Scand. 168 (1), 65–71. 10.1046/j.1365-201x.2000.00660.x 10691781

[B12] Carrasco C. Naziroǧlu M. Rodríguez A. B. Pariente J. A. (2018). Neuropathic pain: delving into the oxidative origin and the possible implication of transient receptor potential channels. Front. Physiol. 9, 95 10.3389/fphys.2018.00095 29491840 PMC5817076

[B13] Chakrabarty A. Liao Z. Smith P. G. (2013). Angiotensin II receptor type 2 activation is required for cutaneous sensory hyperinnervation and hypersensitivity in a rat hind paw model of inflammatory pain. J. Pain 14 (10), 1053–1065. 10.1016/j.jpain.2013.04.002 23726047 PMC3971648

[B14] Chen J. Hackos D. H. (2015). TRPA1 as a drug target--promise and challenges. Naunyn-Schmiedeberg’s Arch. Pharmacol. 388 (4), 451–463. 10.1007/s00210-015-1088-3 25640188 PMC4359712

[B15] Chen J. Kang D. Xu J. Lake M. Hogan J. O. Sun C. (2013). Species differences and molecular determinant of TRPA1 cold sensitivity. Nat. Commun. 4, 2501 10.1038/ncomms3501 24071625 PMC3791479

[B16] Chow B. S. Allen T. J. (2016). Angiotensin II type 2 receptor (AT_2_R) in renal and cardiovascular disease. Clin. Sci. 130 (15), 1307–1326. 10.1042/CS20160243 27358027

[B17] Colloca L. Ludman T. Bouhassira D. Baron R. Dickenson A. H. Yarnitsky D. (2017). Neuropathic pain. Nat Rev Dis Primers 3, 17002 10.1038/nrdp.2017.2 28205574 PMC5371025

[B102] Curato C. Slavic S. Dong J. Skorska A. Altarche-Xifro’ W. Miteva K. (2010). Identification of noncytotoxic and IL-10-producing CD8+AT2R+ T cell population in response to ischemic heart injury. J. Immunol. 185, 6286–6293. 10.4049/jimmunol.0903681 20935205

[B18] Cwynar M. Gąsowski J. Głuszewska A. Królczyk J. Bartoń H. Słowik A. (2016). Blood pressure, arterial stiffness and endogenous lithium clearance in relation to AGTR1 A1166C and AGTR2 G1675A gene polymorphisms. J. Renin-Angiotensin-Aldosterone Syst. 17 (2), 1470320316655669 10.1177/1470320316655669 27339867 PMC5843941

[B19] de Kloet A. D. Pitra S. Wang L. Hiller H. Pioquinto D. J. Smith J. A. (2016). Angiotensin type-2 receptors influence the activity of vasopressin neurons in the paraventricular nucleus of the hypothalamus in male mice. Endocrinology 157 (8), 3167–3180. 10.1210/en.2016-1131 27267713 PMC4967126

[B20] De Logu F. Nassini R. Materazzi S. Gonçalves M. C. Nosi D. Degl’Innocenti D. R. (2017). Schwann cell TRPA1 mediates neuroinflammation that sustains macrophage-dependent neuropathic pain in mice. Nat. Commun. 8 (1), 1887 10.1038/s41467-017-01739-2 29192190 PMC5709495

[B21] Deer T. R. Levy R. M. Kramer J. Poree L. Amirdelfan K. Grigsby E. (2017). Dorsal root ganglion stimulation yielded higher treatment success rate for complex regional pain syndrome and causalgia at 3 and 12 months: a randomized comparative trial. Pain 1584, 669–681. 10.1097/j.pain.0000000000000814 PMC5359787 28030470

[B22] del Camino D. Murphy S. Heiry M. Barrett L. B. Earley T. J. Cook C. A. (2010). TRPA1 contributes to cold hypersensitivity. J. Neurosci. 30, 15165–15174. 10.1523/JNEUROSCI.2580-10.2010 21068322 PMC3021322

[B23] Deshayes F. Nahmias C. (2005). Angiotensin receptors: a new role in cancer?. Trends Endocrinol. Metabol. 16 (7), 293–299. 10.1016/j.tem.2005.07.009 16061390

[B24] Esposito M. F. Malayil R. Hanes M. Deer T. (2019). Unique characteristics of the dorsal root ganglion as a target for neuromodulation. Pain Med. 20 (Suppl. 1), S23–S30. 10.1093/pm/pnz012 PMC6544557 31152179

[B25] Finnerup N. B. Haroutounian S. Kamerman P. Baron R. Bennett D. L. Bouhassira D. (2016). Neuropathic pain: an updated grading system for research and clinical practice. Pain 157 (8), 1599–1606. 10.1097/j.pain.0000000000000492 27115670 PMC4949003

[B26] Fischer T. A. Singh K. O’Hara D. S. Kaye D. M. Kelly R. A. (1998). Role of AT1 and AT2 receptors in regulation of MAPKs and MKP-1 by ANG II in adult cardiac myocytes. Am. J. Physiol. 275 (3), H906–H916. 10.1152/ajpheart.1998.275.3.H906 9724295

[B27] Freynhagen R. Parada H. A. Calderon-Ospina C. A. Chen J. Rakhmawati Emril D. Fernández-Villacorta F. J. (2019). Current understanding of the mixed pain concept: a brief narrative review. Curr. Med. Res. Opin. 35 (6), 1011–1018. 10.1080/03007995.2018.1552042 30479161

[B28] Georgieva D. Georgiev V. (1999). The role of angiotensin II and of its receptor subtypes in the acetic acid-induced abdominal constriction test. Pharmacol. Biochem. Behav. 62, 229–232. 10.1016/s0091-3057(98)00116-6 9972688

[B29] Giorgi S. Nikolaeva-Koleva M. Alarcón-Alarcón D. Butrón L. González-Rodríguez S. (2019). Is TRPA1 burning down TRPV1 as druggable target for the treatment of chronic pain? Int. J. Mol. Sci. 20 (12), 2906 10.3390/ijms20122906 PMC6627658 31197115

[B30] Grace P. M. Gaudet A. D. Staikopoulos V. Maier S. F. Hutchinson M. R. Salvemini D. (2016). Nitroxidative signaling mechanisms in pathological pain. Trends Neurosci. 39 (12), 862–879. 10.1016/j.tins.2016.10.003 27842920 PMC5148691

[B31] Guimond M. O. Gallo-Payet N. (2012). The angiotensin II type 2 receptor in brain functions: an update. Int. J. Hypertens. 2012, 351758 10.1155/2012/351758 23320146 PMC3540774

[B32] Hansen J. L. Servant G. Baranski T. J. Fujita T. Iiri T. Sheikh S. P. (2000). Functional reconstitution of the angiotensin II type 2 receptor and G(i) activation. Circ. Res. 87 (9), 753–759. 10.1161/01.res.87.9.753 11055978

[B33] Hayashida W. Horiuchi M. Dzau V. J. (1996). Intracellular third loop domain of angiotensin II type-2 receptor. Role in mediating signal transduction and cellular function. J. Biol. Chem. 271 (36), 21985–21992. 10.1074/jbc.271.36.21985 8703004

[B34] Heber S. Fischer M. J. M. (2019). Non-analgesic symptomatic or disease-modifying potential of TRPA1. Med. Sci. 7 (10), 99 10.3390/medsci7100099 PMC6836032 31547502

[B35] Hilliard L. M. Jones E. S. Steckelings U. M. Unger T. Widdop R. E. Denton K. M. (2012). Sex-specific influence of angiotensin type 2 receptor stimulation on renal function: a novel therapeutic target for hypertension. Hypertension 59 (2), 409–414. 10.1161/HYPERTENSIONAHA.111.184986 22158645

[B36] Huang X. C. Richards E. M. Sumners C. (1995). Angiotensin II type 2 receptor-mediated stimulation of protein phosphatase 2A in rat hypothalamic/brainstem neuronal cocultures. J. Neurochem. 65 (5), 2131–2137. 10.1046/j.1471-4159.1995.65052131.x 7595499

[B37] IASP Definition of Pain Task Force (2019). IASP’s proposed new definition of pain released for comment. Available at: https://www.iasp-pain.org/PublicationsNews/NewsDetail.aspx?ItemNumber=9218.

[B38] Jones E. S. Vinh A. McCarthy C. A. Gaspari T. A. Widdop R. E. (2008). AT2 receptors: functional relevance in cardiovascular disease. Pharmacol. Ther. 120 (3), 292–316. 10.1016/j.pharmthera.2008.08.009 18804122 PMC7112668

[B39] Kambayashi Y. Bardhan S. Takahashi K. Tsuzuki S. Inui H. Hamakubo T. (1993). Molecular cloning of a novel angiotensin II receptor isoform involved in phosphotyrosine phosphatase inhibition. J. Biol. Chem. 268 (33), 24543–24546. 8227011

[B40] Kang J. Posner P. Sumners C. (1994). Angiotensin II type 2 receptor stimulation of neuronal K+ currents involves an inhibitory GTP binding protein. Am. J. Physiol. 267 (5 Pt 1), C1389–C1397. 10.1152/ajpcell.1994.267.5.C1389 7977700

[B41] Kang J. Richards E. M. Posner P. Sumners C. (1995). Modulation of the delayed rectifier K+ current in neurons by an angiotensin II type 2 receptor fragment. Am. J. Physiol. 268 (1 Pt 1), C278–C282. 10.1152/ajpcell.1995.268.1.C278 7840157

[B42] Kang K. Pulver S. R. Panzano V. C. Chang E. C. Griffith L. C. Theobald D. L. (2010). Analysis of Drosophila TRPA1 reveals an ancient origin for human chemical nociception. Nature 464, 597–600. 10.1038/nature08848 20237474 PMC2845738

[B43] Karnik S. S. Unal H. Kemp J. R. Tirupula K. C. Eguchi S. Vanderheyden P. M. (2015). International union of basic and clinical Pharmacology. XCIX. Angiotensin receptors: interpreters of pathophysiological angiotensinergic stimuli [corrected]. Pharmacol. Rev. 67 (4), 754–819. 10.1124/pr.114.010454 26315714 PMC4630565

[B44] Kaschina E. Namsolleck P. Unger T. (2017). AT2 receptors in cardiovascular and renal diseases. Pharmacol. Res. 125 (Pt A), 39–47. 10.1016/j.phrs.2017.07.008 28694144

[B45] Kemp B. A. Howell N. L. Gildea J. J. Keller S. R. Padia S. H. Carey R. M. (2014). AT₂ receptor activation induces natriuresis and lowers blood pressure. Circ. Res. 115 (3), 388–399. 10.1161/CIRCRESAHA.115.304110 24903104 PMC4116673

[B46] Khan N. Muralidharan A. Smith M. T. (2017). Attenuation of the infiltration of angiotensin II expressing CD3+ T-cells and the modulation of nerve growth factor in lumbar dorsal root ganglia - a possible mechanism underpinning analgesia produced by EMA300, an angiotensin II type 2 (AT2) receptor antagonist. Front. Mol. Neurosci. 10, 389 10.3389/fnmol.2017.00389 29200998 PMC5696600

[B47] Kobayashi K. Fukuoka T. Obata K. Yamanaka H. Dai Y. Tokunaga A. (2005). Distinct expression of TRPM8, TRPA1, and TRPV1 mRNAs in rat primary afferent neurons with adelta/c-fibers and colocalization with trk receptors. J. Comp. Neurol. 493, 596–606. 10.1002/cne.20794 16304633

[B48] Koivisto A. Jalava N. Bratty R. Pertovaara A. (2018). TRPA1 antagonists for pain relief. Pharmaceuticals 11 (4), 117 10.3390/ph11040117 PMC6316422 30388732

[B49] Kumar V. Knowle D. Gavini N. Pulakat L. (2002). Identification of the region of AT2 receptor needed for inhibition of the AT1 receptor-mediated inositol 1,4,5-triphosphate generation. FEBS Lett. 532 (3), 379–386. 10.1016/s0014-5793(02)03713-4 12482596

[B50] Laursen W. J. Anderson E. O. Hoffstaetter L. J. Bagriantsev S. N. Gracheva E. O. (2015). Species-specific temperature sensitivity of TRPA1. Temperature (Austin) 2 (2), 214–226. 10.1080/23328940.2014.1000702 27227025 PMC4843866

[B51] Lemarié C. A. Schiffrin E. L. (2010). The angiotensin II type 2 receptor in cardiovascular disease. J. Renin-Angiotensin-Aldosterone Syst. 11 (1), 19–31. 10.1177/1470320309347785 19861349

[B52] Littlejohn N. K. Keen H. L. Weidemann B. J. Claflin K. E. Tobin K. V. Markan K. R. (2016). Suppression of resting metabolism by the angiotensin AT2 receptor. Cell Rep. 16 (6), 1548–1560. 10.1016/j.celrep.2016.07.003 27477281 PMC4981564

[B53] Lu J. Wu L. Jiang T. Wang Y. Zhao H. Gao Q. (2015). Angiotensin AT2 receptor stimulation inhibits activation of NADPH oxidase and ameliorates oxidative stress in rotenone model of Parkinson's disease in CATH.a cells. Neurotoxicol. Teratol. 47, 16–24. 10.1016/j.ntt.2014.11.004 25446015

[B54] Lum-Naihe K. Toedebusch R. Mahmood A. Bajwa J. Carmack T. Kumar S. A. (2017). Cardiovascular disease progression in female Zucker diabetic fatty rats occurs via unique mechanisms compared to males. Sci. Rep. 7 (1), 17823 10.1038/s41598-017-18003-8 29259233 PMC5736602

[B55] Marion E. Song O. R. Christophe T. Babonneau J. Fenistein D. Eyer J. (2014). Mycobacterial toxin induces analgesia in buruli ulcer by targeting the angiotensin pathways. Cell 157 (7), 1565–1576. 10.1016/j.cell.2014.04.040 24949969

[B56] Mogi M. Iwanami J. Horiuchi M. (2012). Roles of brain angiotensin II in cognitive function and dementia. Int. J. Hypertens. 2012, 169649 10.1155/2012/169649 23304450 PMC3529904

[B57] Mukoyama M. Nakajima M. Horiuchi M. Sasamura H. Pratt R. E. Dzau V. J. (1993). Expression cloning of type 2 angiotensin II receptor reveals a unique class of seven-transmembrane receptors. J. Biol. Chem. 268 (33), 24539–24542. 8227010

[B58] Muralidharan A. Wyse B. D. Smith M. T. (2014). Analgesic efficacy and mode of action of a selective small molecule angiotensin II type 2 receptor antagonist in a rat model of prostate cancer-induced bone pain. Pain Med. 15 (1), 93–110. 10.1111/pme.12258 24433468

[B59] Nemoto W. Ogata Y. Nakagawasai O. Yaoita F. Tadano T. Tan-No K. (2015). Involvement of p38 MAPK activation mediated through AT1 receptors on spinal astrocytes and neurons in angiotensin II- and III-induced nociceptive behavior in mice. Neuropharmacology 99, 221–231. 10.1016/j.neuropharm.2015.07.022 26209257

[B60] Nouet S. Nahmias C. (2000). Signal transduction from the angiotensin II AT2 receptor. Trends Endocrinol. Metabol. 11 (1), 1–6. 10.1016/s1043-2760(99)00205-2 10652498

[B61] Obata K. Katsura H. Mizushima T. Yamanaka H. Kobayashi K. Dai Y. (2005). TRPA1 induced in sensory neurons contributes to cold hyperalgesia after inflammation and nerve injury. J. Clin. Invest. 115, 2393–2401. 10.1172/JCI25437 16110328 PMC1187934

[B62] Patapoutian A. Tate S. Woolf C. J. (2009). Transient receptor potential channels: targeting pain at the source. Nat. Rev. Drug Discov. 8 (1), 55–68. 10.1038/nrd2757 19116627 PMC2755576

[B63] Patel S. N. Fatima N. Ali R. Hussain T. (2020). Emerging role of angiotensin AT_2_ receptor in anti-inflammation: an update. Curr. Pharmaceut. Des. 26 (4), 492–500. 10.2174/1381612826666200115092015 PMC7457547 31939729

[B64] Paulis L. Foulquier S. Namsolleck P. Recarti C. Steckelings U. M. Unger T. (2016). Combined angiotensin receptor modulation in the management of cardio-metabolic disorders. Drugs 76 (1), 1–12. 10.1007/s40265-015-0509-4 26631237 PMC4700059

[B103] Peluso A. A. Bertelsen J. B. Andersen K. Mortsensen T. P. Hansen P. B. Sumners C. (2018). Identification of protein phosphatase involvement in the AT2 receptor-induced activation of endothelial nitric oxide synthase. Clin. Sci. 132 (7), 777–790. 10.1042/CS20171598 29540539

[B65] Pettersson-Fernholm K1 Fröjdö S. Fagerudd J. Thomas M. C. Forsblom C. Wessman M. (2006). The AT2 gene may have a gender-specific effect on kidney function and pulse pressure in type I diabetic patients. Kidney Int. 69 (10), 1880–1884. 10.1038/sj.ki.5000348 16598200

[B66] Pulakat L. Rahman S. Gray A. Knowle D. Gavini N. (2005). Roles of the intracellular regions of angiotensin II receptor AT2 in mediating reduction of intracellular cGMP levels. Cell. Signal. 17 (3), 395–404. 10.1016/j.cellsig.2004.08.007 15567070

[B67] Rice A. S. C. Dworkin R. H. McCarthy T. D. Anand P. Bountra C. McCloud P. I. (2014). EMA401, an orally administered highly selective angiotensin II type 2 receptor antagonist, as a novel treatment for postherpetic neuralgia: a randomised, double-blind, placebo-controlled phase 2 clinical trial. Lancet 383 (9929), 1637–1647. 10.1016/S0140-6736(13)62337-5 24507377

[B68] Rompe F. Unger T. Steckelings U. M. (2010). The angiotensin AT2 receptor in inflammation. Drug News Perspect. 23 (2), 104–111. 10.1358/dnp.2010.23.2.1475901 20369075

[B69] Sakagawa T. Okuyama S. Kawashima N. Hozumi S. Nakagawasai O. Tadano T. (2000). Pain threshold, learning and formation of brain edema in mice lacking the angiotensin II type 2 receptor. Life Sci. 67 (21), 2577–2585. 10.1016/s0024-3205(00)00841-9 11104359

[B70] Sampson A. K. Moritz K. M. Jones E. S. Flower R. L. Widdop R. E. Denton K. M. (2008). Enhanced angiotensin II type 2 receptor mechanisms mediate decreases in arterial pressure attributable to chronic low-dose angiotensin II in female rats. Hypertension 52 (4), 666–671. 10.1161/HYPERTENSIONAHA.108.114058 18711010

[B71] Santabárbara-Ruiz P. López-Santillán M. Martínez-Rodríguez I. Binagui-Casas A. Pérez L. Milán M. (2015). ROS-induced JNK and p38 signaling is required for unpaired cytokine activation during drosophila regeneration. PLoS Genet. 11 (10), e1005595 10.1371/journal.pgen.1005595 26496642 PMC4619769

[B72] Santos R. A. S. Oudit G. Y. Verano-Braga T. Canta G. Steckelings U. M. Bader M. (2019). The renin-angiotensin system: going beyond the classical paradigms. Am. J. Physiol. Heart Circ. Physiol. 316 (5), H958–H970. 10.1152/ajpheart.00723.2018 30707614 PMC7191626

[B73] Santos R. A. S. Sampaio W. O. Alzamora A. C. Motta-Santos D. Alenina N. Bader M. (2018). The ACE2/angiotensin-(1-7)/MAS Axis of the renin-angiotensin system: focus on angiotensin-(1-7). Physiol. Rev. 98 (1), 505–553. 10.1152/physrev.00023.2016 29351514 PMC7203574

[B74] Sawada Y. Hosokawa H. Hori A. Matsumura K. Kobayashi S. (2007). Cold sensitivity of recombinant TRPA1 channels. Brain Res. 1160, 39–46. 10.1016/j.brainres.2007.05.047 17588549

[B75] Sharma N. Belenchia A. M. Toedebusch R. Pulakat L. Hans C. P. (2020). AT2R agonist NP-6A4 mitigates aortic stiffness and proteolytic activity in mouse model of aneurysm. J. Cell Mol. Med. 24, 7393–7404. 10.1111/jcmm.15342 32420690 PMC7339180

[B76] Shepherd A. J. Mickle A. D. Golden J. P. Mack M. R. Halabi C. M. de Kloet A. D. (2018). Macrophage angiotensin II type 2 receptor triggers neuropathic pain. Proc. Natl. Acad. Sci. U. S. A. 115 (34), E8057–E8066. 10.1073/pnas.1721815115 30082378 PMC6112686

[B77] Shepherd A. J. Mohapatra D. P. (2019). Attenuation of unevoked mechanical and cold pain hypersensitivities associated with experimental neuropathy in mice by angiotensin II type-2 receptor antagonism. Anesth. Analg. 128 (6), e84–e87. 10.1213/ANE.0000000000003857 31094778 PMC6652216

[B78] Smith M. T. Anand P. Rice A. S. (2016). Selective small molecule angiotensin II type 2 receptor antagonists for neuropathic pain: preclinical and clinical studies. Pain 157 (Suppl. 1), S33–S41. 10.1097/j.pain.0000000000000369 26785154

[B79] Smith M. T. Wyse B. D. Edwards S. R. (2013). Small molecule angiotensin II type 2 receptor (AT₂R) antagonists as novel analgesics for neuropathic pain: comparative pharmacokinetics, radioligand binding, and efficacy in rats. Pain Med. 14 (5), 692–705. 10.1111/pme.12063 23489258

[B80] St John Smith E. (2018). Advances in understanding nociception and neuropathic pain. J. Neurol. 265 (2), 231–238. 10.1007/s00415-017-8641-6 29032407 PMC5808094

[B81] Steckelings U. M. Kaschina E. Unger T. (2005). The AT2 receptor--a matter of love and hate. Peptides 26 (8), 1401–1409. 10.1016/j.peptides.2005.03.010 16042980

[B82] Steckelings U. M. Kloet A. Sumners C. (2017). Centrally mediated cardiovascular actions of the angiotensin II type 2 receptor. Trends Endocrinol. Metabol. 28 (9), 684–693. 10.1016/j.tem.2017.06.002 PMC5563271 28733135

[B83] Story G. M. Peier A. M. Reeve A. J. Eid S. R. Mosbacher J. Hricik T. R. (2003). ANKTM1, a TRP-like channel expressed in nociceptive neurons, is activated by cold temperatures. Cell 112 (6), 819–829. 10.1016/s0092-8674(03)00158-2 12654248

[B84] Sumners C. Horiuchi M. Widdop R. E. McCarthy C. Unger T. Steckelings U. M. (2013). Protective arms of the renin-angiotensin-system in neurological disease. Clin. Exp. Pharmacol. Physiol. 40 (8), 580–588. 10.1111/1440-1681.12137 23735163

[B85] Sumners C. Peluso A. A. Haugaard A. H. Bertelsen J. B. Steckelings U. M. (2019). Anti-fibrotic mechanisms of angiotensin AT(2) -receptor stimulation. Acta Physiol. 227 (1), e13280 10.1111/apha.13280 30957953

[B86] Toedebusch R. Belenchia A. Pulakat L. (2018). Cell-specific protective signaling induced by the novel AT_2_R-agonist NP-6A4 on human endothelial and smooth muscle cells. Front. Pharmacol. 9, 928 10.3389/fphar.2018.00928 30186168 PMC6111462

[B87] Treede R. D. Jensen T. S. Campbell J. N. Cruccu G. Dostrovsky J. O. Griffin J. W. (2008). Neuropathic pain: redefinition and a grading system for clinical and research purposes. Neurology 70, 1630–1635. 10.1212/01.wnl.0000282763.29778.59 18003941

[B88] Treede R. D. (2018). The International Association for the Study of Pain definition of pain: as valid in 2018 as in 1979, but in need of regularly updated footnotes. Pain Rep 3 (2), e643 10.1097/PR9.0000000000000643 29756089 PMC5902252

[B89] Trevisan G. Benemei S. Materazzi S. De Logu F. De Siena G. Fusi C. (2016). TRPA1 mediates trigeminal neuropathic pain in mice downstream of monocytes/macrophages and oxidative stress. Brain 139 (Pt 5), 1361–1377. 10.1093/brain/aww038 26984186

[B90] Unger T. Steckelings U. M. dos Santos R. A. S. (2015). The protective arm of the renin angiotensin system (RAS): functional aspects and therapeutic implications. London, UK: Academic Press.

[B91] Verbrugghe P. Verhoeven J. Clijsters M. Vervoort D. Schepens J. Meuris B. (2018). The effect of a nonpeptide angiotensin II type 2 receptor agonist, compound 21, on aortic aneurysm growth in a mouse model of marfan syndrome. J. Cardiovasc. Pharmacol. 71 (4), 215–222. 10.1097/FJC.0000000000000560 29300219 PMC5902135

[B92] Vinson G. P. Barker S. Puddefoot J. R. (2012). The renin-angiotensin system in the breast and breast cancer. Endocr. Relat. Canc. 19 (1), R1–R19. 10.1530/ERC-11-0335 22180497

[B93] Wang Y. Del Borgo M. Lee H. W. Baraldi D. Hirmiz B. Gaspari T. A. (2017). Anti-fibrotic potential of AT(2) receptor agonists. Front. Pharmacol. 8, 564 10.3389/fphar.2017.00564 28912715 PMC5583590

[B94] Wei S. G. Yu Y. Zhang Z. H. Weiss R. M. Felder R. B. (2008). Angiotensin II-triggered p44/42 mitogen-activated protein kinase mediates sympathetic excitation in heart failure rats. Hypertension 52 (2), 342–350. 10.1161/HYPERTENSIONAHA.108.110445 18574076 PMC2785116

[B95] Widdop R. E. Jones E. S. Hannan R. E. Gaspari T. A. (2003). Angiotensin AT2 receptors: cardiovascular hope or hype? Br. J. Pharmacol. 140 (5), 809–824. 10.1038/sj.bjp.0705448 14530223 PMC1574085

[B96] Wilson S. R. Gerhold K. A. Bifolck-Fisher A. Liu Q. Patel K. N. Dong X. (2011). TRPA1 is required for histamine-independent, Mas-related G protein-coupled receptor-mediated itch. Nat. Neurosci. 14 (5), 595–602. 10.1038/nn.2789 21460831 PMC3181150

[B97] Xiao L. Haack K. K. Zucker I. H. (2013). Angiotensin II regulates ACE and ACE2 in neurons through p38 mitogen-activated protein kinase and extracellular signal-regulated kinase 1/2 signaling. Am. J. Physiol. Cell Physiol. 304 (11), C1073–C1079. 10.1152/ajpcell.00364.2012 23535237 PMC3677174

[B98] Yang W. Tao Y. Wu Y. Zhao X. Ye W. Zhao D. (2019). Neutrophils promote the development of reparative macrophages mediated by ROS to orchestrate liver repair. Nat. Commun. 10 (1), 1076 10.1038/s41467-019-09046-8 30842418 PMC6403250

[B99] Yvan-Charvet L. Even P. Bloch-Faure M. Guerre-Millo M. Moustaid-Moussa N. Ferre P. (2005). Deletion of the angiotensin type 2 receptor (AT_2_R) reduces adipose cell size and protects from diet-induced obesity and insulin resistance. Diabetes 54 (4), 991–999. 10.2337/diabetes.54.4.991 15793237

[B100] Zhang H. Han G. W. Batyuk A. Ishchenko A. White K. L. Patel N. (2017). Structural basis for selectivity and diversity in angiotensin II receptors. Nature 544 (7650), 327–332. 10.1038/nature22035 28379944 PMC5525545

[B101] Zhang J. Pratt R. E. (1996). The AT2 receptor selectively associates with Gialpha2 and Gialpha3 in the rat fetus. J. Biol. Chem. 271 (25), 15026–15033. 8663053

